# Enhancing cauliflower growth under cadmium stress: synergistic effects of Cd-tolerant *Klebsiella* strains and jasmonic acid foliar application

**DOI:** 10.3389/fmicb.2024.1444374

**Published:** 2024-08-07

**Authors:** Shumila Shahid, Abubakar Dar, Azhar Hussain, Imran Khalid, Muhammad Latif, Hafiz Tanvir Ahmad, Tariq Mehmood, Saud S. Aloud

**Affiliations:** ^1^Department of Soil Science, The Islamia University of Bahawalpur, Bahawalpur, Pakistan; ^2^Department of Extension Education, The Islamia University of Bahawalpur, Bahawalpur, Pakistan; ^3^Department of Agronomy, The Islamia University of Bahawalpur, Bahawalpur, Pakistan; ^4^National Cotton Breeding Institute, The Islamia University of Bahawalpur, Bahawalpur, Pakistan; ^5^Department Sensors and Modeling, Leibniz Institute for Agricultural Engineering and Bioeconomy (ATB), Potsdam, Germany; ^6^Soil Sciences Department, College of Food and Agricultural Sciences, King Saud University, Riyadh, Saudi Arabia

**Keywords:** Cd toxicity, bacterial consortium, jasmonic acid, antioxidants, microbial interaction, immobilization, translocation factor, bioconcentration factor

## Abstract

The pollution of heavy metals (HMs) is a major environmental concern for agricultural farming communities due to water scarcity, which forces farmers to use wastewater for irrigation purposes in Pakistan. Vegetables grown around the cities are irrigated with domestic and industrial wastewater from areas near mining, paint, and ceramic industries that pollute edible parts of crops with various HMs. Cadmium (Cd) is an extremely toxic metal in arable soil that enters the food chain and damages the native biota, ultimately causing a reduction in plant growth and development. However, the use of microbes and growth regulators enhances plant growth and development as well as HM immobilization into the cell wall and hinders their entry into the food chain. Thus, the integrated use of bacterial consortium along with exogenously applied jasmonic acid (JA) mitigates the adverse effect of metal stress, ultimately reducing the metal mobility into roots by soil. Therefore, the current study was conducted to check the impact of Cd-tolerant bacteria and JA on the growth, nutrient status, and uptake of Cd in the cauliflower (*Brassica oleracea*). Our results demonstrated that increasing concentrations of Cd negatively affect growth, physiological, and biochemical attributes, while the use of a bacterial consortium (SS7 + SS8) with JA (40 μmol L^−1^) significantly improved chlorophyll contents, stem fresh and dry biomass (19.7, 12.7, and 17.3%), root length and root fresh and dry weights (28.8, 15.2, and 23.0%), and curd fresh and dry weights and curd diameter (18.7, 12.6, and 15.1%). However, the maximum reduction in soil Cd, roots, and curd uptake was observed by 8, 11, and 9.3%, respectively, under integrated treatment as compared to the control. Moreover, integrating bacterial consortium and JA improves superoxide dismutase (SOD) (16.79%), peroxidase dismutase (POD) (26.96%), peroxidase (POX) (26.13%), and catalase (CAT) (26.86%). The plant nitrogen, phosphorus, and potassium contents were significantly increased in soil, roots, and curd up to 8, 11, and 9.3%, respectively. Hence, a consortium of *Klebsiella* strains in combination with JA is a potential phytostabilizer and it reduces the uptake of Cd from soil to roots to alleviate the adverse impact on cauliflower’s growth and productivity.

## 1 Introduction

Heavy metals (HMs) pose a serious threat to the native biota due to natural and anthropogenic activities all around the globe (Behera et al., 2023). Among abiotic stresses, HMs are one of the biggest threats to arable soils in recent times, which disturb the environment, crop sustainability, and food security (Altaf et al., 2022a; Ghuge et al., 2023). Rapid industrialization and anthropogenic activities, such as the use of inorganic fertilizer, pesticides, and wastewater irrigation for vegetables, are major factors responsible for HM pollution in soil (Meena et al., 2020; Haider et al., 2021). Cadmium (Cd) is a highly toxic metal among all HMs due to its high solubility in water, which can lead to complete crop failure (Sanita di Toppi and Gabbrielli, 1999). Major sources of Cd are mining, electroplating, ceramic, and paint industries, which disturb the environment and whole ecosystem (Villen-Guzman et al., 2021). Moreover, the use of synthetic and phosphatic fertilizers increases the concentration of Cd in soil, air, and water, ultimately increasing the accumulation of Cd in soil (Annar, 2022). However, Cd enters soil by various mechanisms, such as leaching, industrial process, and waste, which are adsorbed by the soil particles. Subsequently, Cd uptake by plants leads to its entry into vital parts, which ultimately destroy plant growth and development (Liu et al., 2021; Khanna et al., 2022). Moreover, the higher amounts of Cd in plants also disturb the CO_2_ absorption rate, photosynthetic rate, and chlorophyll contents (Melki et al., 2022). Cadmium enters the food chain when Cd-contaminated food is consumed by humans, which may cause damage to the human reproductive system, nephrotoxicity, teratogenicity, and endocrine toxicity (Altaf et al., 2022b; Ayub et al., 2023). To remediate these harmful impacts of Cd and all other HMs in the ecosystem, a unique, cheaper, and eco-friendly approach needs to be explored that can reduce Cd uptake from the soil to the root system and enhance the plant tolerance against toxic levels of Cd. Among all recent approaches, bioremediation is the most sustainable and environment-friendly approach to remediate HMs and hinder their entry into the food chain by forming metal complexes and secreting plant growth-promoting substances in the rhizosphere (Kalkan, 2022; Shabaan et al., 2022; Yaashikaa et al., 2022). Moreover, using microbes positively enhances the fertility status of soil and reduces the uptake of Cd in plants (Idris and Yuliar, 2022). The Cd-tolerant bacterial strains sequester Cd in soil through immobilization by secreting polymeric substances, phytohormones, and siderophores. Plant growth-promoting bacteria and phytohormones may form stable complexes with Cd, thus stabilizing it in soil. However, the negative charge of roots may bind with Cd and reduce its uptake in the upper part of the plant. The Cd-tolerant bacterial strains isolated from wastewater include different genera such as *Klebsiella*, *Pseudomonas*, *Enterobacter*, and *Bacillus*, which possess plant growth-promoting abilities and can reduce Cd toxicity (Kamaruzzaman et al., 2020). Thus, the utilization of microbes to ameliorate Cd toxicity for improving environmental quality can be a tool to develop biofertilizers for growing vegetables, fruits, and field crops in contaminated soils.

In addition, exogenous application of hormones, signaling molecules, and osmolytes also gains attention to reduce abiotic stresses (Rizwan et al., 2016a). JA, a signaling molecule and plant growth regulator, acts as a protective agent to boost plant growth and antioxidants during abiotic stress (Mir et al., 2018). The exogenously applied JA binds the various sugars, hydroxylated derivatives, and carbohydrates that accumulate in plant cells in response to various stresses (Ali et al., 2023). The previous study depicted that foliar application of JA decreases the concentration of Cd in rice by reducing the Cd translocation from roots to grain (Li et al., 2022). When the concentration of HMs exceeds in plants, it produces reactive oxygen species (ROS) and causes oxidative stress in response to HM toxicity. However, JA scavenges the overproduction of ROS and reduces the oxidative damage caused by HMs (Foyer and Noctor, 2011). Moreover, the previous studies also depicted that JA can significantly increase the activities of antioxidant enzymes to boost the defense mechanism against HM stress in plants (Kamran et al., 2021). Manzoor et al. (2022) stated that the exogenously applied JA enhanced the growth of *Pisum sativum* L. cultivars by enhancing photosynthetic pigments and antioxidant efficiency. The individual application of Cd-tolerant bacteria and JA has been reported in the literature to alleviate Cd toxicity under normal and stressed conditions (Ali et al., 2018; Nazli et al., 2020), but no study is available on the combined effect of both stress alleviator JA and metal-tolerant bacteria. Our previous study demonstrated that the combined application of a Cd-tolerant bacterial consortium along with JA foliar application significantly improved cauliflower growth and immobilized the soil Cd in plant roots (Shahid et al., 2023). Here, we hypothesized that a Cd-tolerant bacterial consortium in combination with JA subsequently alleviates the Cd-induced adverse impact on the cauliflower crop. Moreover, co-application of the Cd-tolerant bacterial consortium with JA could be more efficient as compared to sole application to alleviate the Cd stress by regulating the antioxidant defense system in cauliflower. Thus, the current study has been planned to address the synergistic effects of Cd-tolerant *Klebsiella* strains and JA foliar application to enhance the growth of cauliflower plants with the following objectives: (i) to examine the role of synergistic effects of Cd-tolerant *Klebsiella* strains and JA in mitigating the adverse impact of Cd on the growth and development of the cauliflower crop and (ii) to evaluate the potential use of bacterial strains and JA in reducing the entry of Cd into plants.

## 2 Materials and methods

### 2.1 Collection of bacterial strains and inoculation of cauliflower seeds

A pot trial was conducted to check the efficacy of plant growth-promoting Cd-tolerant bacterial strains in combination with JA to ameliorate Cd toxicity, promote growth under Cd toxicity, regulate the antioxidant defense system, and improve nutrient efficiency. Pre-isolated and pre-characterized Cd-tolerant bacterial strains *Klebsiella* sp. (SS7) and *Klebsiella pneumoniae* (SS8) with accession nos. MW829780 and MW829781, respectively, were used in this study and screened for plant growth-promoting characteristics in our earlier study (Shahid et al., 2023). The experiment was performed in the Department of Soil Science, Institute of Soil and Water Resources warehouse, the Islamia University of Bahawalpur. Cauliflower seeds of the latest variety (HS-65), widely accepted in the farmer’s community, were obtained from the Oilseeds Research Station, Bahawalpur. The seeds were disinfected with a 2% sodium hypochlorite solution. The inoculum was prepared by growing bacterial strains SS7 and SS8 separately in Dworkin and Foster (DF) minimal salt media, which is composed of sucrose (10 g), K_2_HPO_4_ (2.5 g), KH_2_PO_4_ (2.5 g), (NH_4_)_2_HPO_4_ (1.0 g), MgSO_4_.7H_2_O (0.2 g), FeSO_4_.7H_2_O (0.01 g), MnSO_4_.7H_2_O (0.007 g), and agar powder (15 g). The media was incubated at 30 ± 1°C in a shaking incubator at 100 rpm for 48 h (Model SI9R-2, Shellab, Riverside, OH, USA). After incubation, the bacterial cells were harvested by centrifugation at 22°C for 20 min at 9000 rpm (Model: UNIVERSAL 320R, Hettich, Germany). Furthermore, the pallets were dissolved in sterilized distilled water after repeated washing, and the final culture was prepared by taking equal proportions (v/v) from both strains in sterile flasks and vortexed for 1 min for consortium application.

#### 2.1.1 Soil sampling and pot trial

The soil sample was collected from the research area of the Department of Soil Science at the Islamia University of Bahawalpur and analyzed for various physicochemical properties of soil following a standard protocol by Ryan et al. (2001), as given in Table 1. Moreover, the soil was analyzed for HM contamination by using the atomic absorption spectrophotometer (AAS) model AAS-240FS, Agilent, Santa Clara, CA, USA, following the standard protocol of Hseu et al. (2002). The pot experiment was conducted in the wire house by carefully managing the natural growth conditions, ambient light, and 10–12°C temperature. For soil contamination, CdCl_2_ salt at 150 mg kg^−1^ was used and mixed in soil 2 weeks before the experiment. Approximately 10 kg of air-dried soil was filled in each pot with three replications, and 10 inoculated and un-inoculated cauliflower seeds were sown in each pot. Surface sterilized cauliflower seeds were inoculated by dipping them in a broth mixture for half an hour before sowing. The pots were arranged as a completely randomized design (CRD) with factorial arrangements. Ten days after germination, the extra plants were removed by thinning, and a uniform population was maintained. The plant nutrients requirement was fulfilled by applying the recommended doses of P and K (90 and 60 kg ha^−1^) by using sulfate of potash (SOP) and diammonium phosphate (DAP) at the time of sowing, while a recommended dose of nitrogen (N) (120 kg ha^−1^) using urea was applied in three splits after 20 days of germination. After 3 weeks of sowing, only one plant of the cauliflower crop was maintained in the pot and a foliar application of JA was applied. A solution of JA was prepared by dissolving it in 100 μL of absolute ethanol (Thaler et al., 1999). A stock solution of 1 mM JA was prepared in a 1-L volumetric flask, and the working solution of JA (40 μmol L^−1^) was prepared by diluting the stock solution as described by Bano et al. (2021). After 3 weeks of sowing, 40 μmol L^−1^ JA was sprayed with a nozzle sprayer (5 mL per plant) to the respective treatments and repeated every week.

**Table 1:** Physiochemical properties of soil used in the pot experiment.

Physiochemical properties	Values
Soil texture	Sandy clay loam
ECe	1.64 dSm^−1^
pH	7.9
Saturation percentage	33%
Organic matter	0.49%
Nitrogen	0.05%
Phosphorus	5.69 mg kg^−1^
Potassium	87 mg kg^−1^
Cadmium	1.11 mg kg^−1^
Lead	0.42 mg kg^−1^
Nickel	ND
Iron	35 mg kg^−1^
Zinc	38 mg kg^−1^

### 2.2 Plant parameters

#### 2.2.1 Cauliflower growth and yield attributes

At physiological maturity, plants were analyzed for physiological attributes such as chlorophyll content (SPAD value). Furthermore, at the time of harvesting after 35 days of sowing, the length of roots and stems and the fresh weight of roots and stems of cauliflower plants were measured using an electrical balance and meter rod. The growth parameters were recorded for each plant in every treatment, and the mean values were determined in triplicate. After that, the roots of cauliflower plants were washed using distilled water and oven-dried at 70°C for 48 h to check dry weight (Rizwan et al., 2019).

### 2.3 Antioxidant activity

The fresh leaf sample (0.5 g) was taken in a pre-cooled mortar on ice. The sample was dissolved in 4 mL of pre-cooled phosphate buffer solution [Na_2_HPO.12H_2_O (16.385 g) + NaH_2_PO_4_.2H_2_O (0.663 g)], and the solution was made up to 1,000 mL by adding distilled water and maintained at a pH of 7.8. After homogenization of the sample on ice, phosphate buffer solution (4 mL) was added and centrifuged for 20 min at 4°C at 10,000 rpm, and the supernatant was collected to determine the different antioxidants such as catalase (CAT), superoxide dismutase (SOD), and peroxidase (POX). For the determination of SOD and POD, a modified nitro blue tetrazolium (NBT) indicator was used, as described by Beauchamp and Fridovich (1971). In addition, the decomposition of H_2_O_2_ at 240 nm was observed to measure the CAT activity, Whereas the POX activity was measured by mixing phosphate buffer and guaiacol with enzymes extract and reading was taken at 470 nm an ultraviolet–visible (UV–VIS) spectrophotometer (Model; Carry 60; Agilent, Santa Clara, CA, USA) (Chance and Maehly, 1995).

Proline contents in the curd of cauliflower were determined by using a 0.5-g fresh leaf sample. Sulphosalicylic acid (3%) was used to process the sample in flasks according to the following procedure by Bates et al. (1973). The contents were filtered by using a Whatman No. 2 after homogenization and adding glacial acetic acid and ninhydrin. The flask was cooled, and the contents were extracted using toluene. The absorbance was measured by using a spectrophotometer at 520 nm, and a standard curve was prepared for the proline concentration (Bates et al., 1973).

### 2.4 Mineral analysis

For mineral analyses, a dry plant sample of 0.2 g with 6 mL of concentrated H_2_SO_4_ was mixed and left overnight. In addition, 1 mL of H_2_O_2_ was also used, and the solution was heated at 300°C on a hot plate for 1 h until the color of the sample was transparent. The samples were analyzed for total macronutrients (NPK) analysis by using the standard protocol, as N was determined by Kjeldahl distillation and titration with 0.01 N H_2_SO_4_, P was determined by the yellow method on a spectrophotometer, and K was determined using a flame photometer (Ryan et al., 2001). Moreover, for the determination of Fe and Zn, the method of extraction by diethylenetriaminepentaacetic acid (DTPA) was used. The reading was determined by using an atomic absorption spectrophotometer (model AAS-240 FS, Agilent, USA). The Fe and Zn in soil were calculated by a standard calibration curve (Lindsay et al., 1978).

### 2.5 Determination of Cd concentration

For Cd determination, 1 g of air-dried soil was weighed, and digestion proceeded at 300°C, as followed by Allen and Rae (1986). After a clear solution, the samples were filtered and diluted with distilled water. The Cd concentration in plants and soil was measured by using an atomic absorption spectrophotometer (AAS) model AAS-240 FS, Agilent, USA.

### 2.6 Statistical analysis

The data were evaluated for significance by using the CRD with factorial arrangement to construct a two-way analysis of variance (ANOVA) of the results (Steel et al., 1997), and the means were compared by using the least significant difference test (LSD). Excel (MS Office) was used to calculate the standard error, mean, and graphs.

## 3 Results

### 3.1 Agronomic parameters

A pot trial was conducted to check the synergistic effects of Cd-tolerant *Klebsiella* strains and JA foliar application under Cd stress. The use of integrating bacterial consortium and JA significantly enhanced the growth and yield attributes of the cauliflower crop under Cd stress. A significant decrease in physiological and growth parameters of the cauliflower crop under Cd stress without amendment of Cd-tolerant bacterial strains and JA was observed. Furthermore, the application of Cd-tolerant bacterial strains in combination with JA significantly enhanced the growth attributes under Cd stress. The combined application of Cd-tolerant bacterial consortium and JA was significantly efficient as compared to individual applications under Cd toxicity. The bacterial consortium (SS7 + SS8) in combination with JA significantly improved cauliflower leaf chlorophyll content by 19.8% as compared to the control treatment. The same treatment also significantly improved (*p* < 0.05) the stem fresh weight (12.7%) and stem dry weight (17.3%) of cauliflower as compared to the control treatment (Table 2). Moreover, the cauliflower root length (28.8%), root fresh weight (15.2%), and root dry weight (23.0%) were also significantly enhanced by 28.8, 15.2, and 23.0%, respectively, as compared to the control treatment (Table 3). The yield parameters of cauliflower were also significantly enhanced by the application of consortium with JA under Cd stress, and the maximum increase in fresh weight of curd (8.68%), dry weight of curd (12.6%), and curd diameter (15.2%) was 8.68, 12.6, and 15.2%, respectively, as compared to the control treatments (*p* = 0.0316) (Table 4). However, individual application of the Cd-tolerant bacterial consortium significantly improved stem dry weight by 12.7%, root dry weight by 17.3%, plant height by 8%, and curd dry weight by 8.5% as compared to the control treatment. Moreover, the foliar application of JA significantly improved chlorophyll contents by 15.1% as compared to other treatments under Cd stress. However, a bacterial consortium in combination with JA improved chlorophyll content by 29.7%, stem fresh weight by 14.3%, stem dry weight by 19.9%, root fresh weight by 19.2%, root dry weight by 22.1%, curd fresh weight by 29.6%, and curd dry weight by 12.6% under normal soil in the absence of Cd stress as compared to the sole application of treatments (*p* < 0.05).

**Table 2:** Effect of Cd-tolerant bacterial consortium in combination with JA on chlorophyll content, stem fresh weight, and stem dry weight of cauliflower in a pot trial.

Treatment	Chlorophyll		Stem fresh weight (g)		Stem dry weight (g)	
	0 mg Cd kg^−1^	150 mg Cd kg^−1^	0 mg Cd kg^−1^	150 mg Cd kg^−1^	0 mg Cd kg^−1^	150 mg Cd kg^−1^
Control	37 ± 0.6^d^	29 ± 0.3^f^	42 ± 0^c^	37 ± 0.3^e^	8.8 ± 0.2^c^	6.1 ± 0.3^e^
SS7 + SS8	41 ± 0.7^c^	31 ± 0.3^f^	45 ± 0.3^b^	38 ± 0.3^d^	9.6 ± 0.1^b^	6.3 ± 0.2^de^
JA	44 ± 0.6^b^	33 ± 0.1^f^	46 ± 0.6^b^	40 ± 0.3^d^	9.6 ± 0.1^b^	6.7 ± 0.3^de^
SS7 + SS8 + JA	48 ± 1.5^a^	34 ± 0.3^e^	47 ± 0.6^a^	41 ± 0.3^c^	10.5 ± 0.3^a^	7.2 ± 0.2^d^
**LSD (*p* ≤ 0.05)**	**2.14**		**1.46**		**0.76**	

The superscript letter also tell us about the significance of the treatments as treatments with different letters are significantly differ from each other.

**Table 3:** Effect of Cd-tolerant bacterial consortium in combination with JA on root fresh weight, root dry weight, and root length of cauliflower in a pot trial.

Treatment	Root fresh weight (g)		Root dry weight(g)		Root length (cm)	
	0 mg Cd kg^−1^	150 mg Cd kg^−1^	0 mg Cd kg^−1^	150 mg Cd kg^−1^	0 mg Cd kg^−1^	150 mg Cd kg^−1^
Control	32 ± 0.2^c^	25 ± 0.3^f^	7.2 ± 0.2^bc^	5.5 ± 0.3^d^	12 ± 0.2^c^	8.6 ± 0.3^f^
SS7 + SS8	35 ± 0.7^b^	26 ± 0.2^ef^	7.5 ± 0.3^b^	5.9 ± 0.4^d^	12 ± 0.4^bc^	10.5 ± 0.3^de^
JA	35 ± 0.3^b^	26 ± 0.3^e^	7.9 ± 0.1^b^	5.8 ± 0.2^d^	13 ± 0.1^b^	10.1 ± 0.2^e^
SS7 + SS8 + JA	38 ± 0.3^a^	28 ± 0.3^d^	8.8 ± 0.2^a^	6.8 ± 0.1^c^	14 ± 0.3^a^	11.1 ± 0.2^d^
**LSD (*p* ≤ 0.05)**	**1.21**		**0.67**		**0.87**	

The superscript letter also tell us about the significance of the treatments as treatments with different letters are significantly differ from each other.

**Table 4:** Effect of Cd-tolerant bacterial consortium in combination with JA on curd fresh weight, curd dry weight, and curd diameter of cauliflower plants in a pot trial.

Treatment	Curd fresh weight (g)		Curd dry weight (g)		Curd diameter (mm)	
	0 mg Cd kg^−1^	150 mg Cd kg^−1^	0 mg Cd kg^−1^	150 mg Cd kg^−1^	0 mg Cd kg^−1^	150 mg Cd kg^−1^
Control	215 ± 3.5^c^	175 ± 2.1^e^	56 ± 0.3^c^	49 ± 0.2^f^	14 ± 0.3^b^	11 ± 0.6^d^
SS7 + SS8	253 ± 11.8^b^	187 ± 1.5^de^	59 ± 0.3^b^	52 ± 0.3^de^	15 ± 0.3^ab^	12 ± 0.7^cd^
JA	257 ± 1.8^b^	192 ± 3.6^d^	58 ± 0.1^b^	51 ± 0.6^e^	15 ± 0.6^ab^	12 ± 0.4^cd^
SS7 + SS8 + JA	276 ± 3.1^a^	196 ± 3.0^d^	63 ± 1.2^a^	54 ± 0.3^d^	16 ± 0.6^a^	13 ± 0.3^c^
**LSD (*p* ≤ 0.05)**	**14.1**		**1.7**		**1.6**	

The superscript letter also tell us about the significance of the treatments as treatments with different letters are significantly differ from each other.

### 3.2 Antioxidants

**Figure 1:**
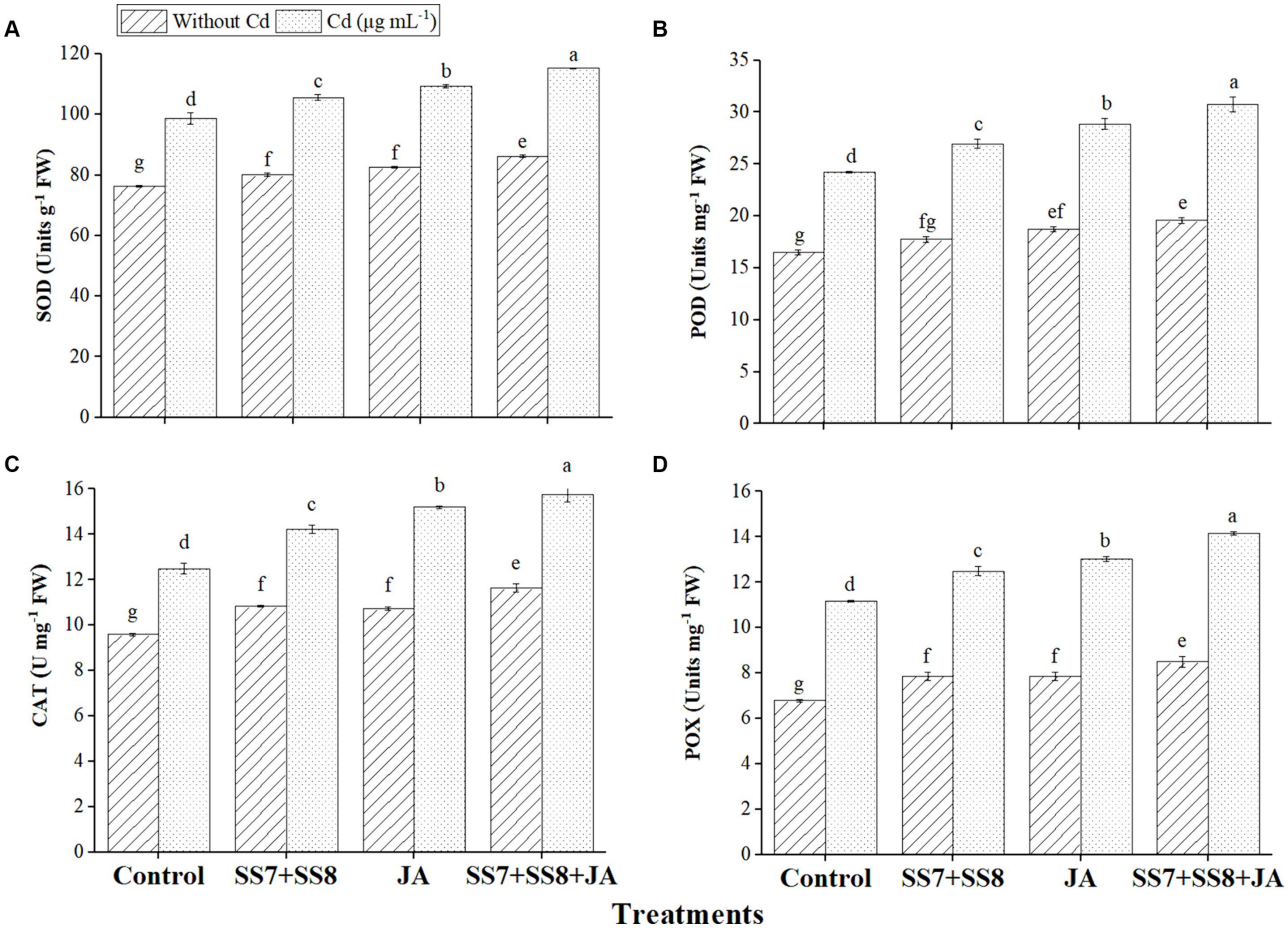
Effect of integrating bacterial consortium with jasmonic acid on **(A)** SOD (*p* = 0.0021); **(B)** POD (*p* = 0.7621); **(C)** CAT (*p* = 0.0001); and **(D)** POX (*p* = 0.0012) activity of a cauliflower plant under Cd stress in a pot trial.

The data regarding the antioxidant enzyme activities of cauliflower plants showed that SOD, POD, CAT, and POX activities were significantly increased under Cd stress. The inoculation of the bacterial consortium (SS7 + SS8) resulted in a greater increase in SOD by 11.5%, POD by 12.5%, CAT by 13.8%, and POX by 15.9% under Cd stress as compared to the control treatment (*p* < 0.05). In addition, individual application of JA significantly enhanced SOD (10.8%), POD (19.1%), POX (21.6%), and CAT (30.2%) activities as compared to the control treatment (*p* < 0.05) (Figure 1). Contrastingly, the combined application of bacterial consortium (SS7 + SS8) and foliar application of JA stimulated the increased antioxidant activity to minimize ROS-induced damage under Cd toxicity, as SOD, POD, POX, and CAT activities were improved by 16.8, 26.9, 26.1, and 26.9%, respectively (*p* < 0.05) (Figure 1).

### 3.3 Efficacy of bacterial consortium and JA on the mineral concentration in cauliflower

**Figure 2:**
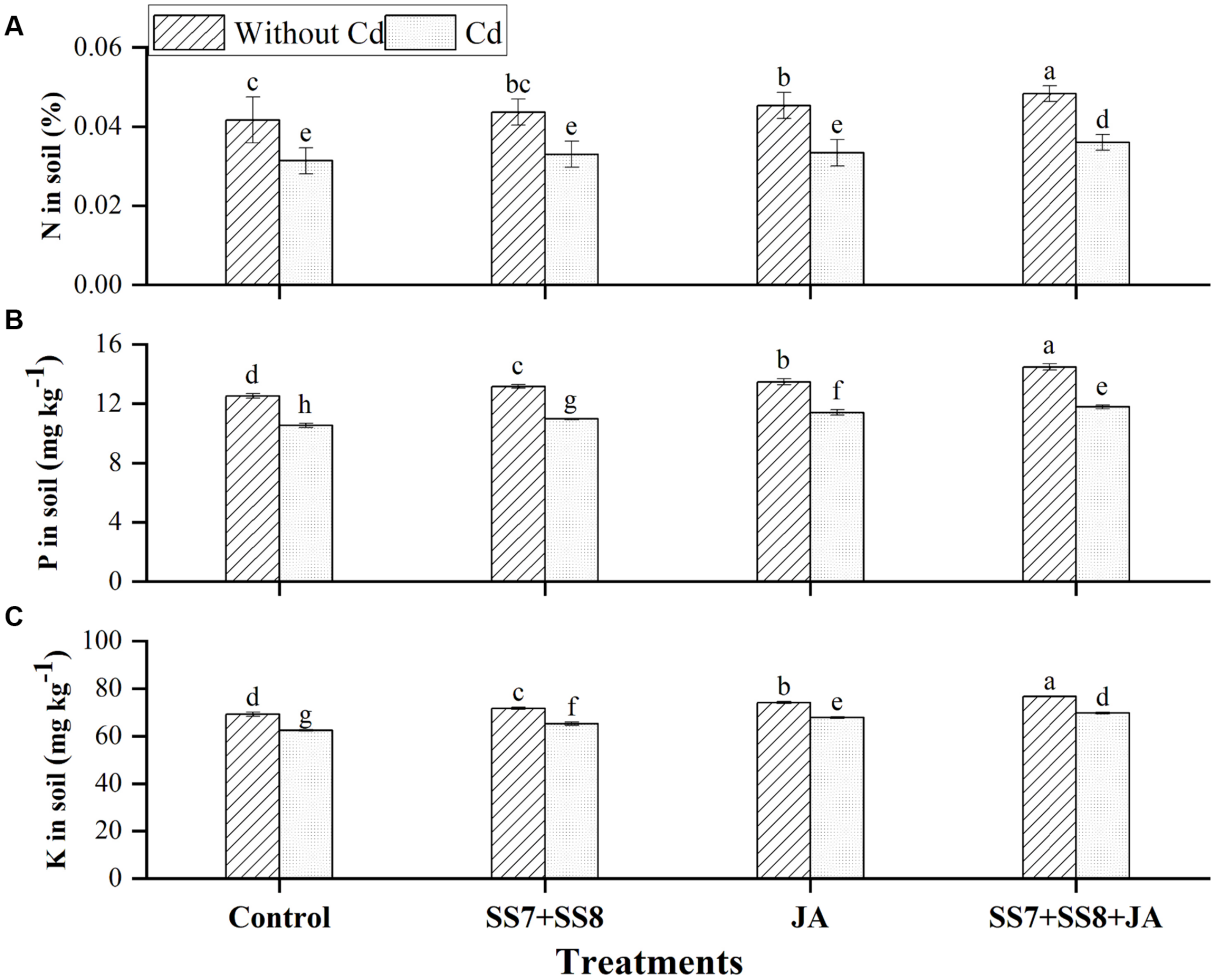
Effect of integrating bacterial consortium with jasmonic acid on **(A)** N in soil (*p* = 0.0001); **(B)** P in soil (*p* = 0.2568); and **(C)** K in soil (*p* = 1.2413) under Cd stress in a pot trial.

**Figure 3:**
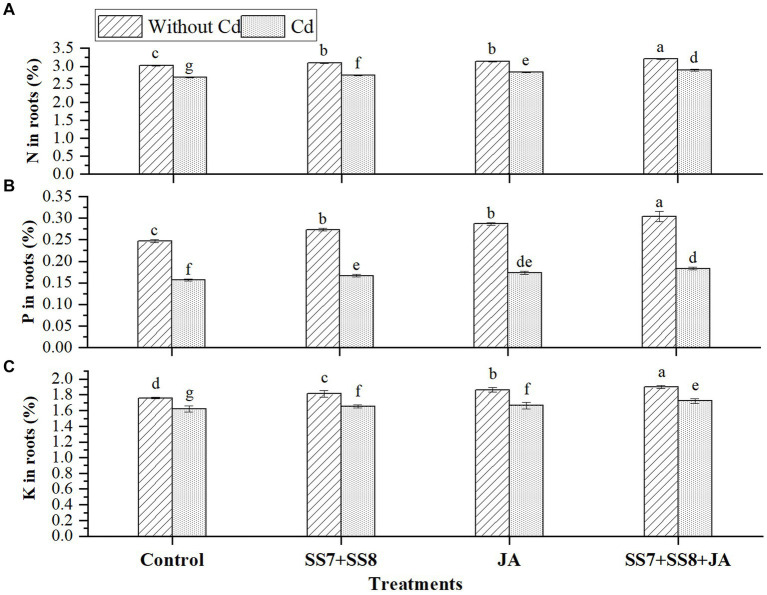
Effect of integrating bacterial consortium with jasmonic acid on **(A)** N in roots (*p* = 0.0444); **(B)** P in roots (*p* = 0.0166); and **(C)** K in roots (*p* = 0.0308) of cauliflower plant under Cd stress in a pot trial.

The combined application of bacterial consortium and JA improved the macronutrients in soil and cauliflower plant in the current study. The Cd-tolerant bacterial consortium in combination with JA led to a substantial production of N, P, and K contents under Cd stress as compared to the control treatments (Figure 2). Moreover, a bacterial consortium in combination with JA significantly improved N, P, and K in soil by 14.9, 11.7, and 11.7%, respectively, under Cd stress as compared to the control treatment. However, in the absence of Cd toxicity, bacterial consortium in combination with JA increased N, P, and K in soil by 16.8, 15.6, and 10.9%, respectively, as compared to the control treatment (Figure 2). Additionally, a significant increase in mineral content was also observed in the roots of cauliflower plants, where N, P, and K contents were significantly improved by 7.6, 17.0, and 10.4%, respectively, under Cd stress (*p* < 0.05) (Figure 3). Moreover, individual application of Cd-tolerant bacteria improved N, P, and K in roots by 5, 10, and 8.5%, respectively, as compared to the control treatments.

**Figure 4:**
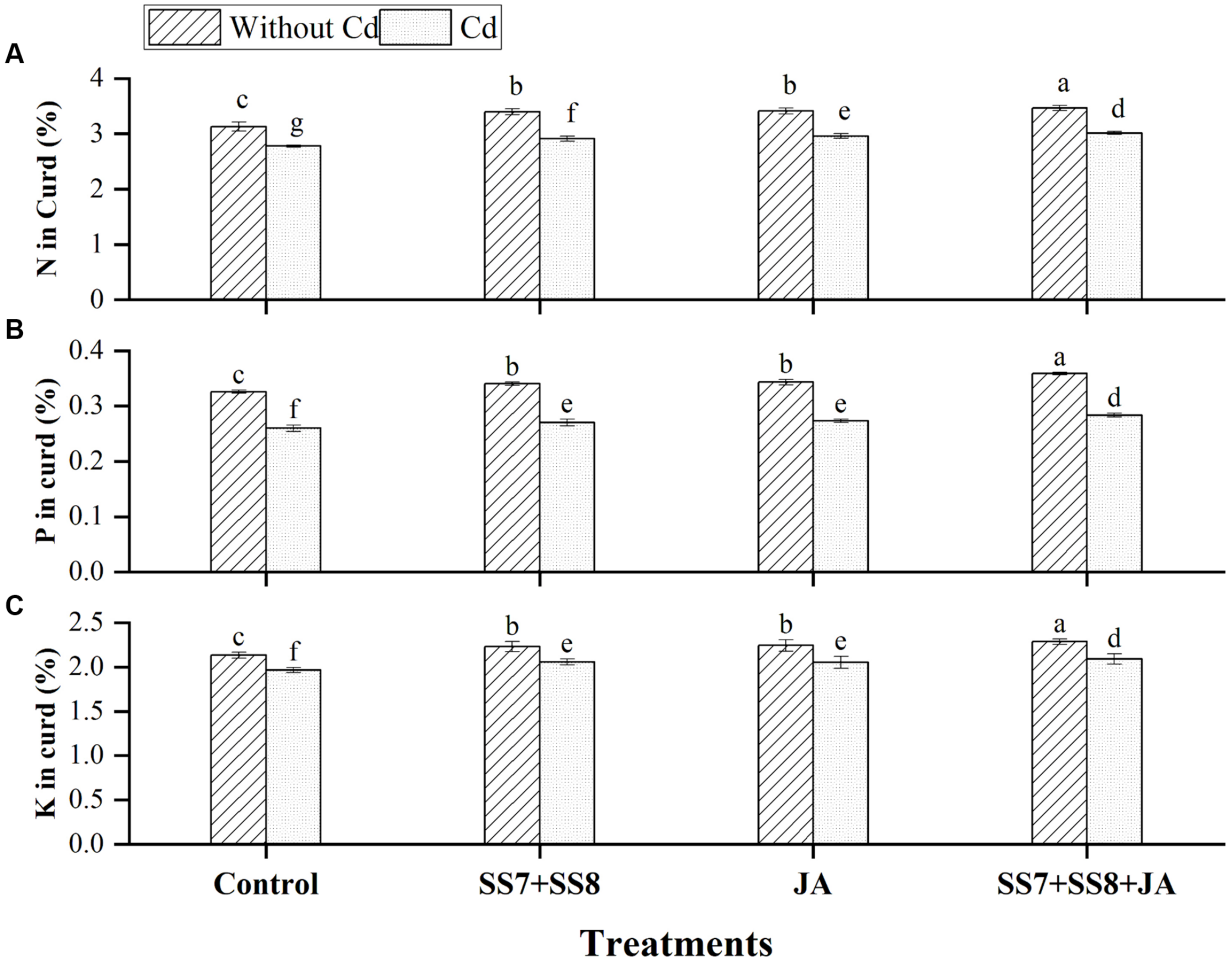
Effect of integrating bacterial consortium with jasmonic acid on **(A)** N in curd (*p* = 0.0435); **(B)** P in curd (*p* = 0.0167); and **(C)** K in curd (*p* = 0.0331) of cauliflower plant under Cd stress in a pot trial.

In addition, under Cd stress, a maximum concentration of N, P, and K was also observed in the curd of cauliflower, where N, P, and K contents were increased by 9.5, 10.0, and 6.27%, respectively, as compared to the un-inoculated control treatment. In addition, individual application of Cd-tolerant bacterial consortium significantly improved N by 6.6%, P by 5.8%, and K by 5.2%, respectively, in curd as compared to the control treatment under Cd stress. However, the application of bacterial consortium with JA significantly increased N, P, and K contents of curd by 10.7, 10.0, and 7.2%, respectively, compared to the control treatment (*p* < 0.05) (Figure 4).

### 3.4 Cd concentration in soil and cauliflower plant

**Figure 5:**
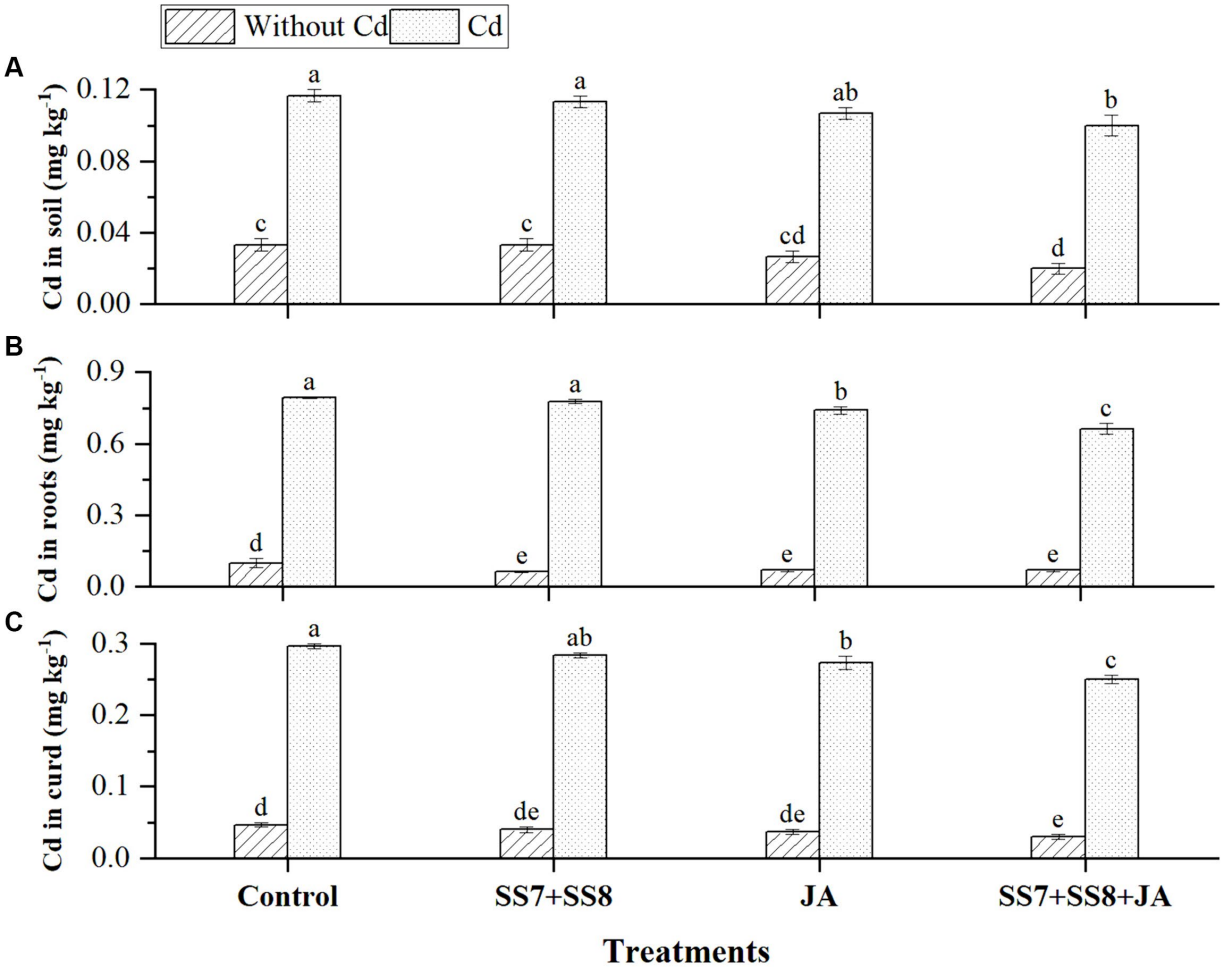
Effect of integrating bacterial consortium with jasmonic acid decreases **(A)** Cd in soil (*p* = 0.0112); **(B)** Cd in roots (*p* = 0.0244); and **(C)** Cd in curd (*p* = 0.0138) of cauliflower plant under Cd stress in a pot trial.

**Figure 6:**
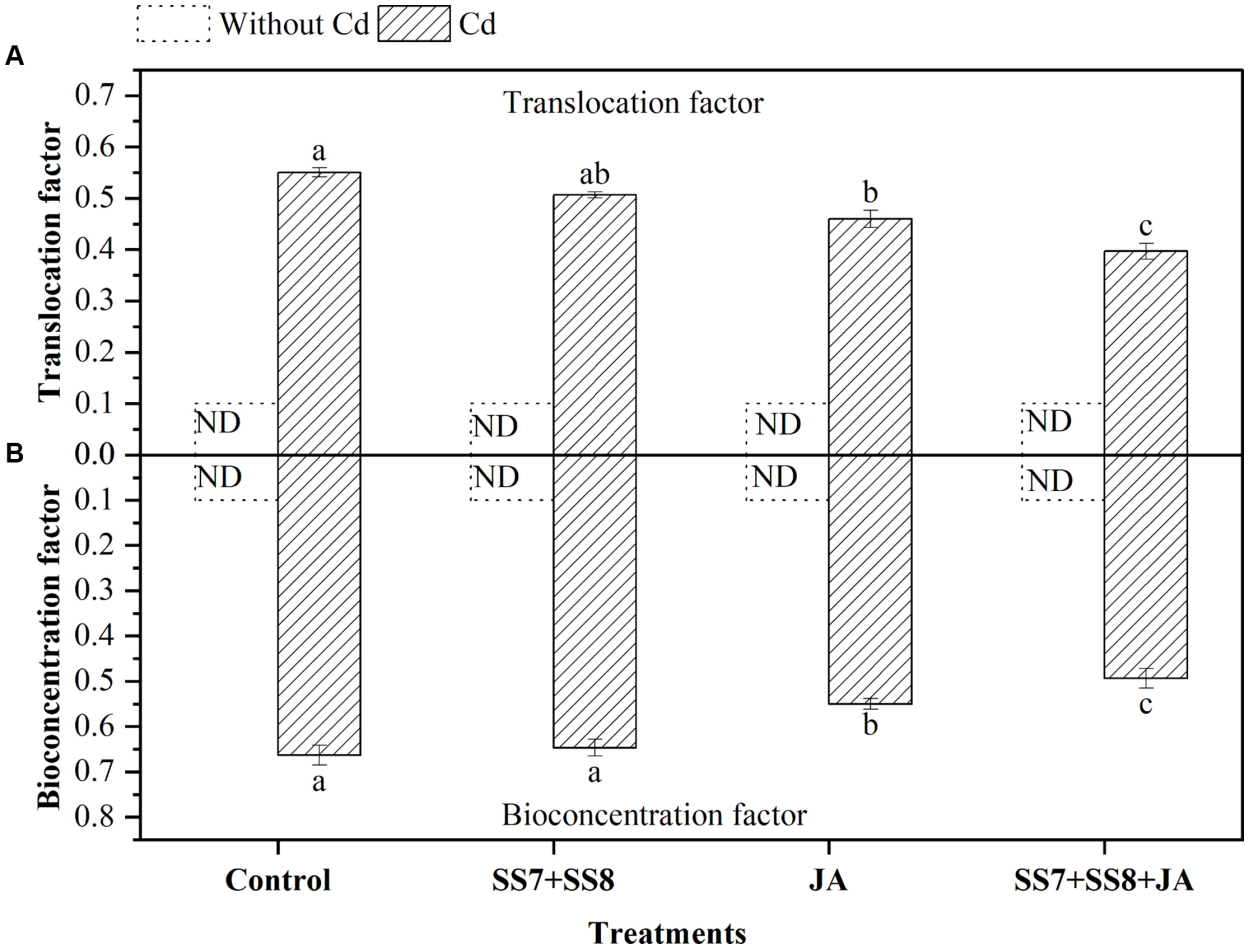
Effect of integrating bacterial consortium with jasmonic acid on **(A)** translocation factor (*p* = 0.0001) and **(B)** bioconcentration factor (*p* = 0.0009) of Cd under Cd stress in a pot trial.

The bacterial consortium in combination with JA application resulted in a remarkable reduction in the concentration of Cd in soil and different parts of the cauliflower plant (Figure 5). The maximum reduction in Cd concentration was measured in soil by 8%, roots by 11.5%, and curd by 9.3% as compared to the individual application of amendments (*p <* 0.05). However, individual application of Cd-tolerant bacterial consortium also reduced Cd concentration by 6.2, 5.8, and 8.5% in soil, roots, and curd, respectively (*p* < 0.05). Moreover, in the absence of Cd stress, the concentration of Cd in cauliflower plants was not detected. Simultaneously, as compared to other treatments, Cd-tolerant bacterial strains in combination with JA inhibit Cd translocation from soil to plant parts by 7.5%. Bioconcentration factors were also reduced by the immobilization of Cd by Cd-tolerant bacterial consortium under Cd stress (Figure 6).

### 3.5 Yield parameter of curd

**Figure 7:**
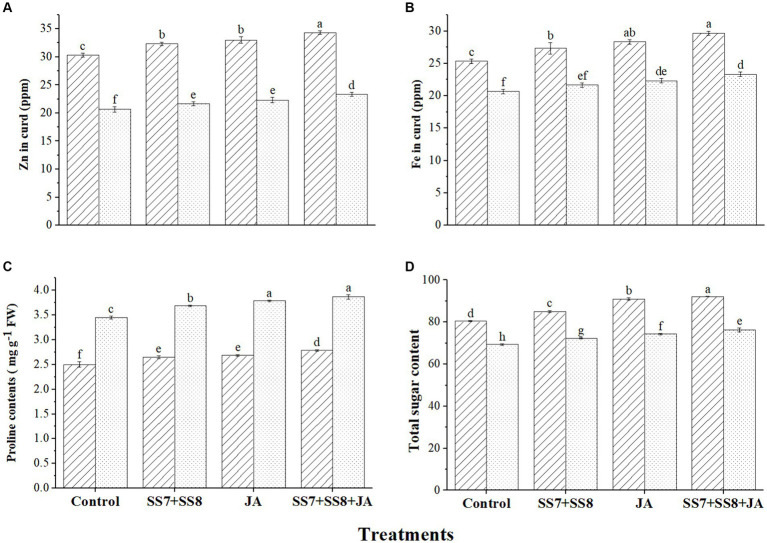
Effect of integrating bacterial consortium with jasmonic acid on **(A)** Zn in curd (*p* = 0.0021); **(B)** Fe in curd (*p* = 0.0001); **(C)** proline contents (*p* = 0.0974); and **(D)** total sugar content (*p* = 0.1224) of cauliflower plant under Cd stress in a pot trial.

The application of bacterial consortium and JA significantly improved yield parameters and Zn, Fe, proline, and total sugar content in the curd of cauliflower and resulted in higher micronutrient efficiencies (Figure 7). The maximum biofortification of Zn in cauliflower curd was observed under treatment with the bacterial consortium and JA, which was 12.9% higher under Cd stress compared to the control treatment. Similarly, a significant increase was also observed for Fe (15.1%), proline (12.1%), and total sugar content (10.1%) by integrating the bacterial consortium with JA under Cd stress as compared to the control treatment. However, individual application of bacterial consortium also increased the fortification and osmolyte concentrations in cauliflower curd, as Zn contents were increased by 6.82%, Fe contents by 5.28%, proline contents by 6.75%, and sugar contents by 5.78% as compared to the un-inoculated control treatment. However, foliar application of JA also increased Zn, Fe, proline, and sugar contents by 5.84, 5.01, 5.23, and 4.98%, respectively, as compared to the control treatments.

### 3.6 Pearson’s correlation

**Figure 8:**
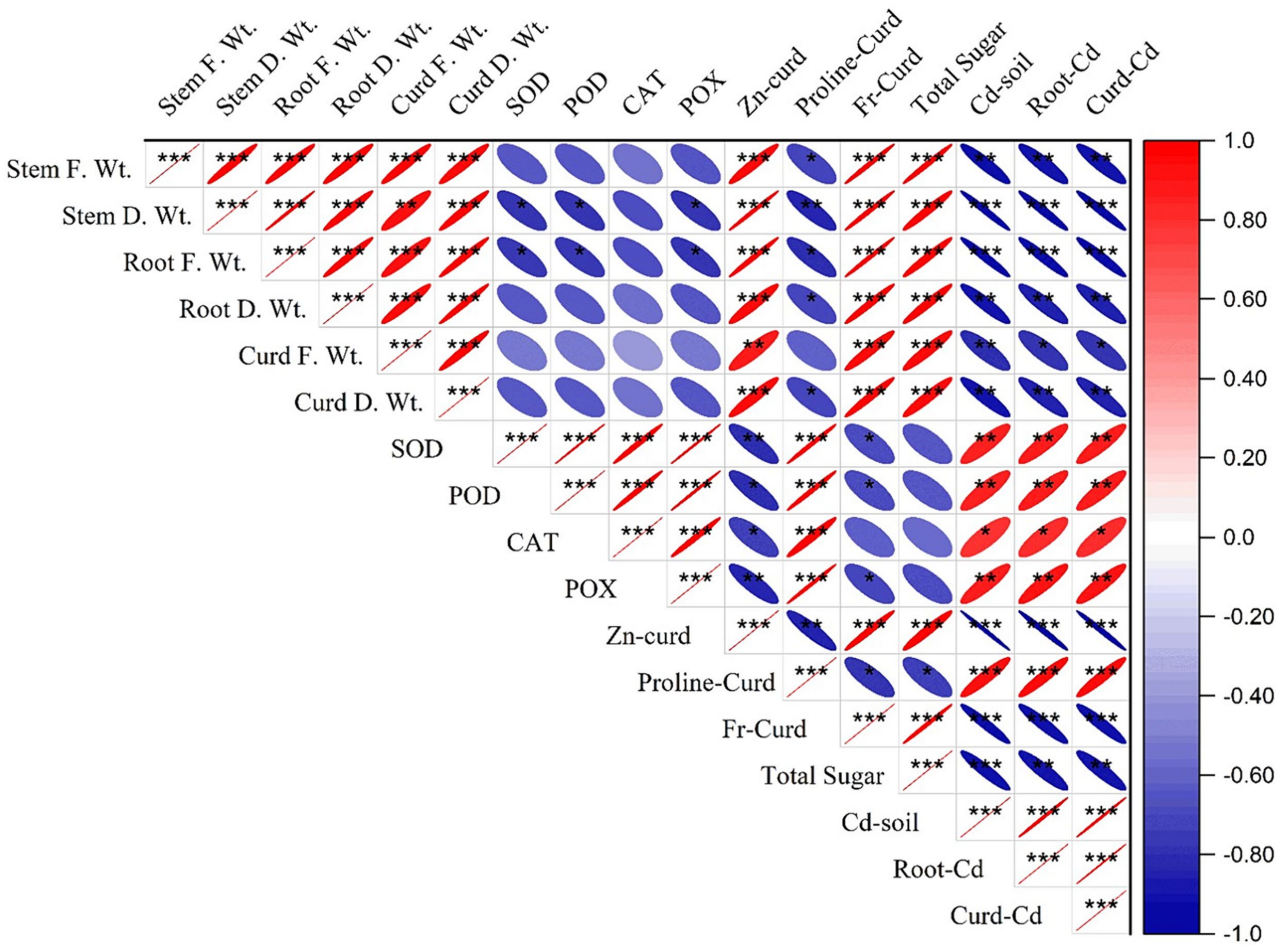
Correlation of Cd concentration in plants, with its different growth and physiological parameters. *p<0.05, **p<0.01, ***p<0.001.

Pearson’s correlation described the relation between different parameters of the study. The results presented in Figure 8 depict an inverse relationship between the plant stress indicator (proline) and the growth and yield parameters and a direct relationship with Cd concentration in soil, root, and curd. This indicates that the uptake of cadmium increased the proline contents in plant as a stress indicator. Similar tendencies for antioxidants with growth and yield attributes and Cd concentration were found. Hence, the integration of bacterial consortium and JA application regulated the physiological stress indicators and antioxidants to cope with the Cd stress and sustain the growth and development of cauliflower.

## 4 Discussion

HM stress has interfered with the productivity and sustainability of agriculture around the world. However, global resources have not maintained the same pace as the population (Grant, 2018). Moreover, the use of synthetic fertilizers, paints, mining, and ceramic industries has increased the concentration of Cd to exploit the whole ecosystem and environment (Hayat et al., 2019). The Cd concentration in Pakistani soil is more than 184 mg kg^−1^ and increasing day by day due to anthropogenic sources and this is an alarming situation as the threshold level is approximately 100 mg kg^−1^ for arable soils in Pakistan (Waseem et al., 2014; Asgher et al., 2015). However, the resources are insufficient to upgrade and meet the demands of the population. To combat all the nutritional and sustainable requirements of the global population, alternate and eco-friendly strategies need to be explored to immobilize and phytostabilize the metal in soil (Haider et al., 2021). In this regard, to mitigate the adverse effects of HMs, plant-growth-promoting metal-tolerant bacterial strains and plant growth regulators play an important role in mitigation strategies due to their environmental-friendly and cost-effective nature in the environment. These metal-tolerant bacterial strains utilize carbon in their niche as a food source, ultimately improving plant growth. These PGPRs bind the various functional groups on their surface, ultimately sequestering and immobilizing metal to reduce toxicity (Shabaan et al., 2022). Moreover, exogenously applied growth regulators play a significant role in crop physiology and stress defense in plants, ultimately mitigating the abiotic stresses (Rizwan et al., 2016b). Hence, the current study examined the individual and combined application of a Cd-tolerant integrating bacterial consortium in combination with JA on growth and yield attributes, production of antioxidants, mineral analysis, and Cd content in cauliflower under Cd stress.

In the current study, a reduction in growth attributes was observed due to Cd stress, which might have decreased the potential use of nutrients and water (Ahmad et al., 2015). A significant decline was observed in chlorophyll content due to the uptake of Cd in the cauliflower plant due to higher stress-generating ethylene levels, which might be a reason for the significant reduction in growth and chlorophyll content. Glick et al. (1999) reported that ethylene production under stress conditions induced a negative and harmful impact on crops’ productivity. Matile et al. (1997) also reported the mechanism by which, under stress conditions, ethylene accumulates in plants and promptly decomposes lipids in the cell wall. When lipids degrade in plants, ethylene comes into contact with chlorophyllase genes and activates the chloroplast, which ultimately degrades chlorophyll, resulting in poor photosynthesis and chlorophyll content. The chlorophyll content was significantly improved with the application of bacterial consortium and plant growth regulator due to less accumulation of ethylene by the treatments applied (Danish et al., 2020). These PGPRs secrete IAA and ACC deaminases that cleave ethylene into ammonia and are utilized as nitrogen sources by PGPRs (Zafar-Ul-Hye et al., 2020). The findings were also supported by Dutta et al. (2018), who reported a reduction in root and shoot lengths due to Cd stress in *Brassica juncea* L. Moreover, we observed that Cd reduced the length and dry weight of shoots and roots due to a reduction in photosynthesis activity and restricted water and mineral uptake by roots (Hasan et al., 2020). However, the literature also supported and demonstrated that under Cd stress, the root and shoot length of *Vigna mungo* reduced due to the uptake of Cd (Rizvi and Khan, 2018). The reason for the Cd-induced decline in plant growth is attributed to photosynthetic activity and restricted water and mineral uptake by the roots (Sharma et al., 2020). We observed a synergistic effect of the dual combination of *Klebsiella* strains with the foliar application of JA, which significantly improves plant growth by enhancing photosynthetic activity and root proliferation. Moreover, due to the synergistic effect, a Cd-tolerant bacterial consortium in combination with JA binds the Cd by immobilizing its mobility into plants via roots and restricting entry to the aerial part of the plant. Naveed et al. (2020) and Bali et al. (2018) reported that the Cd-tolerant bacterial strains and foliar application of JA mitigate Cd, Cu, and Pb stress in *Arabidopsis thaliana* and tomato plants. The effective use of Cd-tolerant bacterial strains also improves the growth and development of plants, as described in previous studies (Shahid et al., 2023).

Under HM stress, the overproduction of ROS and H_2_O_2_ damages the plant cell and membrane structure; however, the production of antioxidants in response to abiotic stress is a significant indicator of stress tolerance (Kusvuran, 2021; Rolon-Cardenas et al., 2022). Our results demonstrated that the combined application of bacterial consortium and JA increased the antioxidant status in cauliflower plant leaves compared to the control treatments under Cd toxicity. Mir et al. (2018) also reported that the use of bacterial consortium and foliar application of JA enhanced the higher production of antioxidant enzymes such as SOD, POD, CAT, and POX under Cd stress. Several studies have shown that foliar application of JA could regulate the redox potential of plants. Kamran et al. (2021) also reported that under Cd and Cr stress, the level of antioxidant status increased under different vegetable crops with the application of JA. The study is also in line with Pramanik et al. (2017), who reported the positive effect of inoculation on antioxidant status by using *Klebsiella pneumoniae* strain K5 under Cd stress.

The rate of Cd accumulation in plant parts from soil varies and is transported from soil to roots depending on plant species and the available soil fraction. In the current study, the bacterial consortium reduced the uptake of Cd from soil to roots and decreased the Cd accumulation by foliar application of JA on the surface of leaves during growth stages. A similar study was also reported by Zeng et al. (2022) that Cd-tolerant bacterial strains reduced the Cd uptake by immobilization in the soil, thus enhancing the sequestration of Cd in soil by producing polymeric substances, phytohormones, and siderophores.

Moreover, the reduction in Cd concentration in cauliflower was associated with the ability of bacterial consortium and foliar application of JA. The use of bacterial consortium binds the Cd by chelating agents in soil and makes it less available for plant uptake. The translocation factor indicates the ability of Cd transportation from one tissue to the next, while the bioconcentration factor indicates the ability of Cd accumulation by cauliflower. In the current study, the Cd-tolerant bacterial consortium inhibits Cd uptake from soil to roots. In contrast, foliar application of JA inhibits Cd uptake from the roots to the curd of cauliflower.

Under stress conditions, plants use different adjustments to overcome the negative impact of environmental stresses in which proline and other metabolites accumulate in plants and act as protective agents (Nadeem et al., 2015; Tanveer et al., 2022). Irfan et al. (2014) and Ahmad et al. (2019) reported higher production of proline levels under Cd stress by different plants. When proline and other metabolites are produced in plants, they boost metabolic energy by adjusting intracellular osmotic potential and regulating the metabolic process, thus protecting the plant against abiotic stresses (Rai et al., 2003; Silveira et al., 2003). Our study revealed that Cd-tolerant bacterial strains with the potential for EPS production enhance the level of proline due to a reduction in the negative effects of Cd, which is also supported by Khan and Bano (2019). The cauliflower curd was also evaluated for mineral content, and the results revealed that the combined application of bacterial consortium and JA significantly improved Fe, Zn, and total sugar content in the curd as compared to the control treatment. The results are also in line with the studies by Hussain et al. (2011) and Anwar et al. (2022), who reported a significant increase in nutrient contents in soil, roots, and curd of cauliflower plant. In the current study, pre-identified Cd-tolerant bacterial strains *Klebsiella* sp. (SS7) and *Klebsiella pneumoniae* (SS8) and foliar application of JA were examined under pot conditions to check their efficacy for the productivity of the cauliflower crop. However, the dual functionality of integrating the bacterial consortium in combination with JA significantly improved overall results as compared to the un-inoculated control treatment. Thus, bacterial consortium along with JA can be used as a potential biofertilizer to promote productivity on a sustainable basis.

## 5 Conclusion

HM pollution caused by urbanization and industrialization poses a serious threat to vegetables, oilseed crops, and fodder. It also causes detrimental effects to humans when HMs enter the food chain. In the current study, the dual application of a Cd-tolerant integrating bacterial consortium and JA seemed to be a viable option to improve plant growth and antioxidants and reduce the uptake of Cd under Cd stress. Moreover, it also plays a significant role in detoxifying the metal from soil and improving plant growth in metal-stress conditions. Hence, the use of bacterial consortiums and growth regulators could be further enhanced, and it is needed to evaluate their efficacy to reduce the toxicity of other metals in various vegetable crops for sustainable crop production.

## Data availability statement

The datasets presented in this study can be found in online repositories. The names of the repository/repositories and accession number(s) can be found at: https://www.ncbi.nlm.nih.gov/, accession numbers: MW829780 and MW829781.

## Author contributions

SS: Conceptualization, Data curation, Formal analysis, Methodology, Writing – original draft, Writing – review & editing, Software. AD: Conceptualization, Data curation, Formal analysis, Methodology, Software, Validation, Writing – original draft, Writing – review & editing, Visualization. AH: Conceptualization, Data curation, Formal analysis, Funding acquisition, Methodology, Resources, Supervision, Validation, Writing – original draft, Writing – review & editing. IK: Investigation, Writing – review & editing, Software. ML: Supervision, Validation, Writing – review & editing. HA: Validation, Writing – review & editing, Data curation, Visualization. TM: Validation, Writing – review & editing, Data curation, Visualization. SA: Funding acquisition, Validation, Writing – review & editing.
